# Supplementary material for: Evaluation of Antioxidant Activity and Biotransformation of Opuntia Ficus Fruit: The Effect of In Vitro and Ex Vivo Gut Microbiota Metabolism
Source: Molecules. 2022 Nov 4;27(21):7568. doi: 10.3390/molecules27217568 (PMC9653959; doi:10.3390/molecules27217568)
Supplement: Supplementary file 1 [file molecules-27-07568-s001.zip › molecules-1912763-supplementary.pdf]

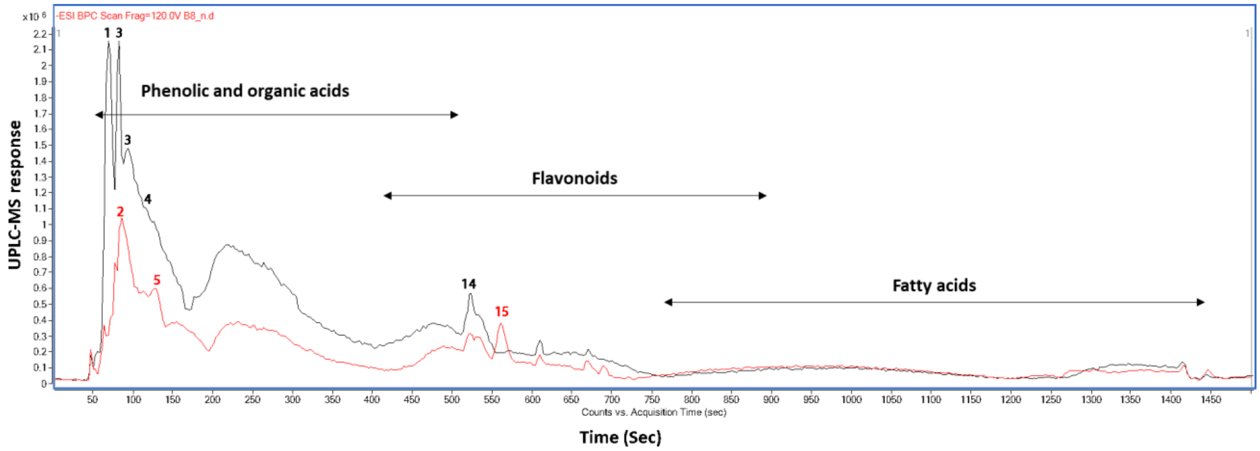

Figure S1. Representative UHPLC-QTOF-MS chromatogram for the negative ionization mode of *O. ficus* methanolic extract incubated with the selected microbial strains at a concentration of 5 mg/ml after 0.5 h (black) and 24 h (red), both chromatograms are characterized by three regions; (50-500 s for phenolic and organic acids, (400-900 s) for flavonoids, and (750-1450 s) for fatty acids

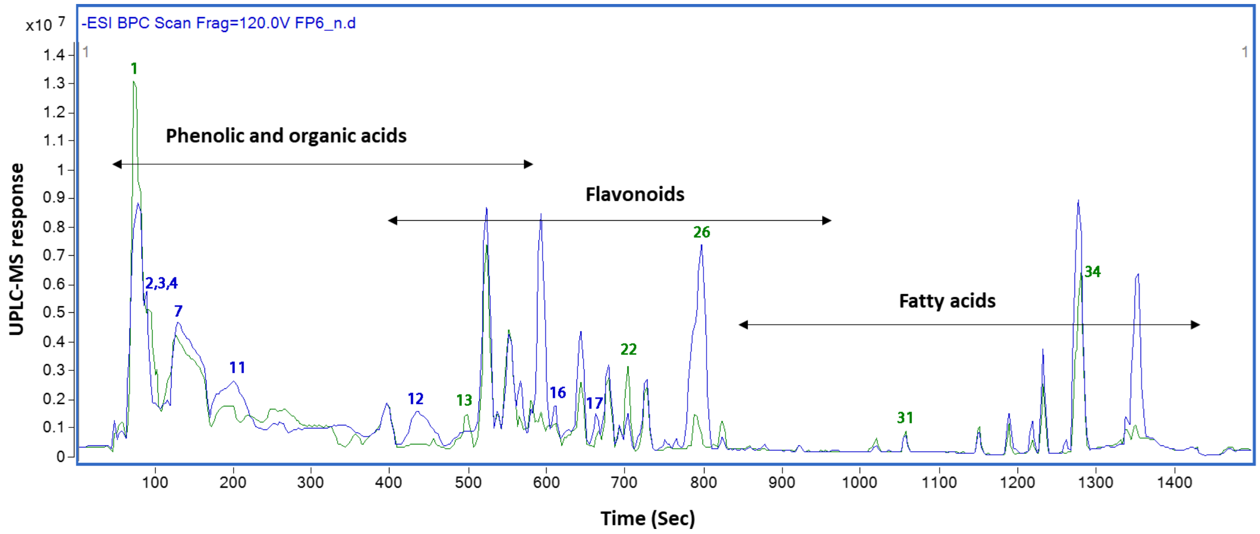

Figure S2. Representative UHPLC-QTOF-MS chromatogram for the negative ionization mode of untreated *O. ficus* methanolic extract (green) and treated *ex-vivo* (blue) with bacterial culture isolated from actual fecal matter at a concentration of 10 mg/ml, both chromatograms are characterized by three regions; (50-530 s for phenolic and organic acids, (400-1000 s) for flavonoids, and (710-1370 s) for fatty acids

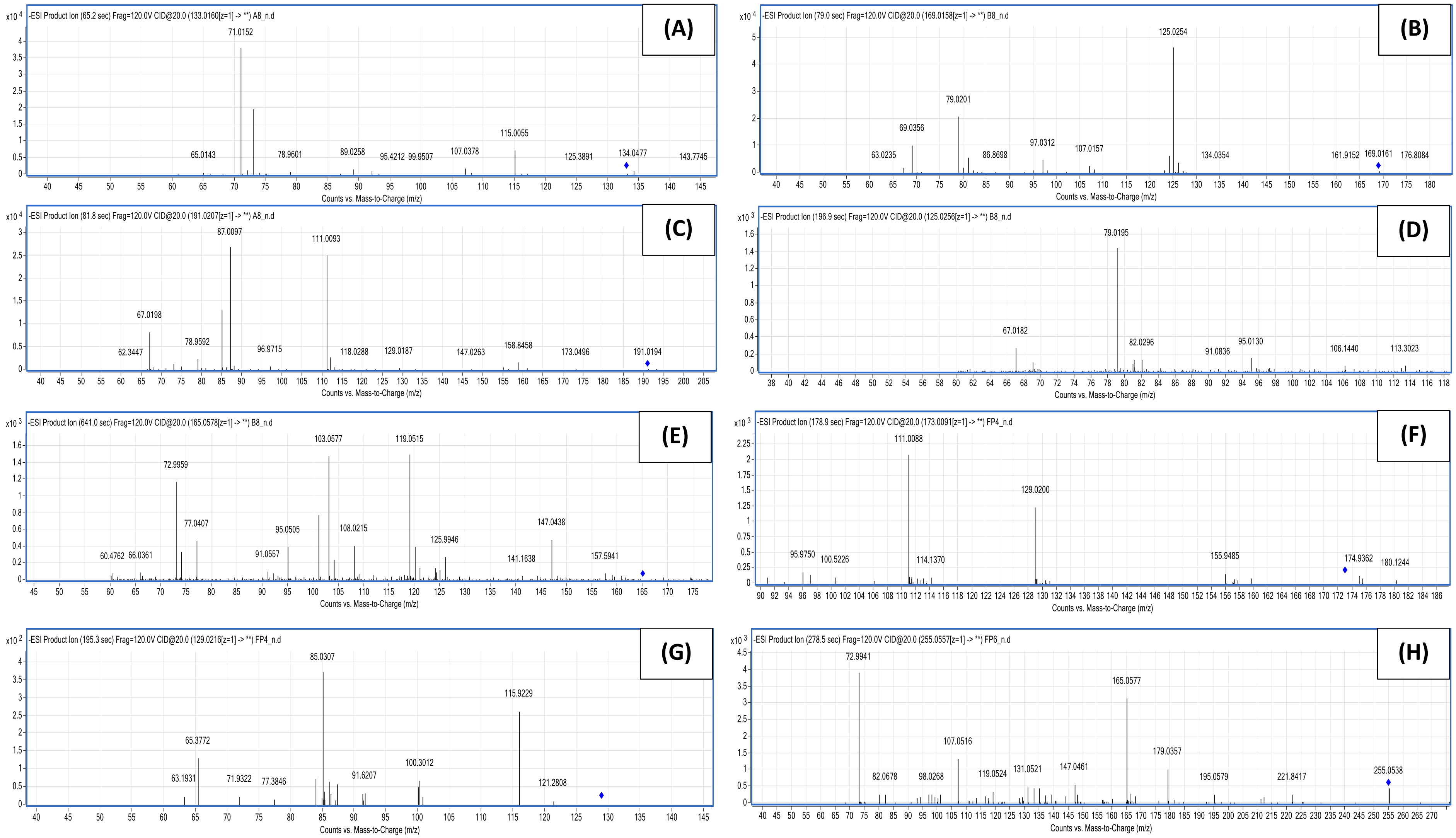

Figure S3. Tandem mass spectral data of some of the major metabolites studied in in vitro and ex vivo assays namely; A) Malic acid, B) Gallic acid, C) (iso)Citric acid, D) Phloroglucinol, E) 3-(4-Hydroxyphenyl) propanoic acid, F) Aconitic acid, G) Mesaconic acid, H) Piscidic acid.

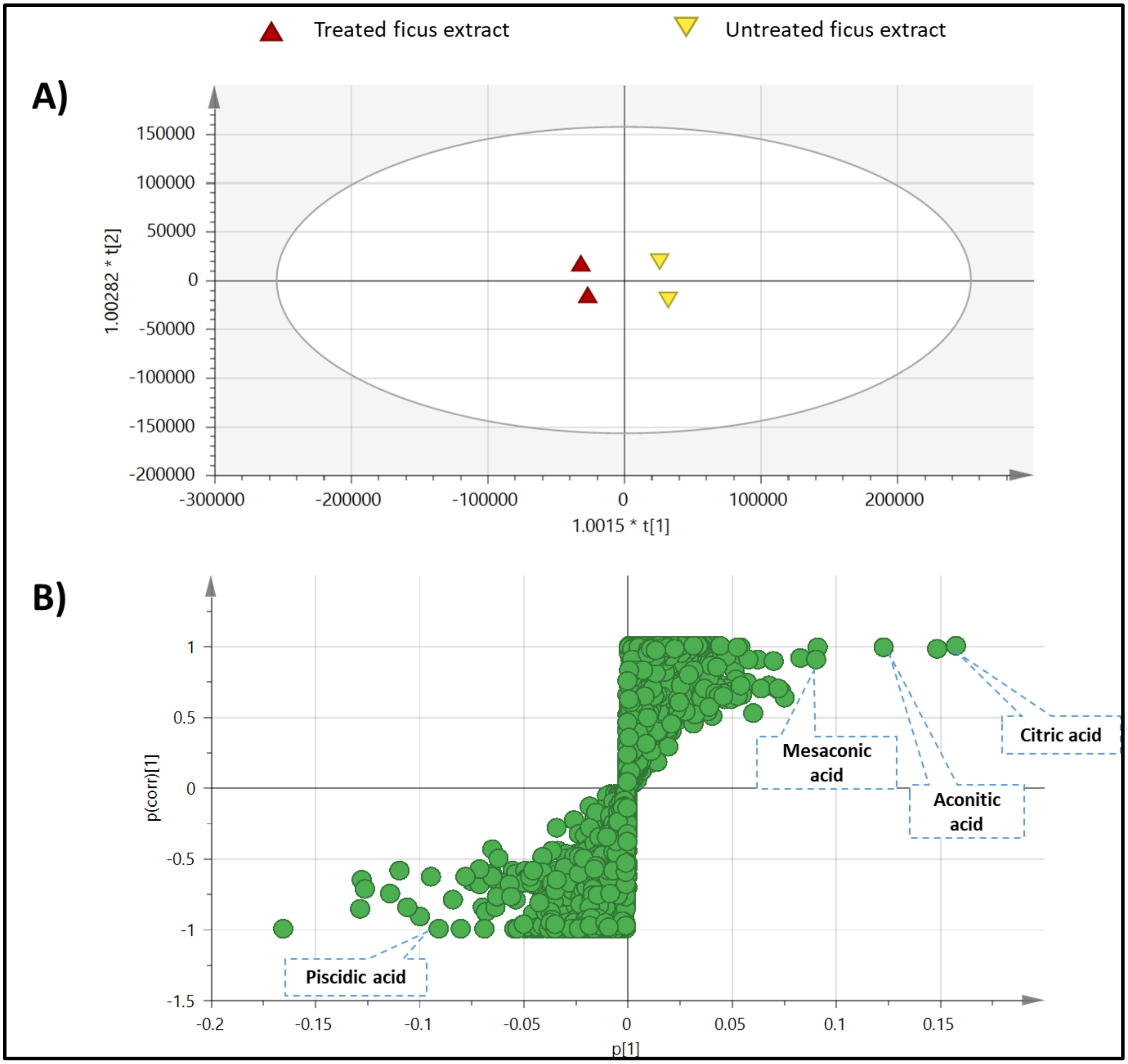

Figure S4. (A) OPLS model of *O. ficus* treated *ex-vivo* with gut microbiota culture isolated form fecal matter based on treatment; untreated samples (yellow) modeled against treated (red) B) S-plot of OPLS model, metabolites with positive p[1] values indicates higher abundance in untreated sample mainly; (iso)citric, aconitic and mesaconic acids, while negative p[1] indicates higher abundance within treated sample mainly; piscidic acid.

Table S1. Metabolites identified in *O. ficus* samples treated with gut microbiota at two time intervals; 0.5 and 24 h along with their relative abundance. Results are expressed as relative percentile (average ± std deviation, n=3) of the total peak areas of identified metabolites.

Peak No.	[M-H]$^-$	Name	Abundance % at 0.5 h	Abundance % at 24 h
**1**	133.0154	Malic acid*	9.38±0.57	2.09±1.06
**2**	169.0161	Gallic acid*	9.2±2.85	14.2±0.36
**3**	189.0057	(iso)Citrate*	50.79±2.11	40.12±1.96
**4**	207.0159	Hydroxycitric acid*	1.69±0.11	1.32±0.38
**5**	125.0256	Pyrogallol*	0	4.57±0.73
**6**	117.0204	Succinic acid*	7.22±1.32	12.43±3.81
**7**	125.0257	Phloroglucinol*	0	2.01±0.81
**8**	205.0368	Homocitric acid*	2.52±0.18	0
**9**	153.0214	Protocatechuic acid	0.11±0.04	0
**10**	199.0265	Fumarylacetoacetic acid (Maleylacetoacetic acid)*	0.8±0.06	3.15±0.33
**11**	117.0566	Hydroxyvaleric acid*	1.11±0.77	1.95±0.17
**12**	541.2307	Isorhamnetin glycoside*	2.06±0.4	1.69±0.17
**13**	219.0532	Dimethyl citrate	9.45±0.42	8.45±0.5
**14**	183.032	Methyl gallate	1.54±0.03	1.01±0.04
**15**	165.0585	3-(4-Hydroxyphenyl) propanoic acid*	0	3.57±0.704
**16**	563.1102	Kaempferol*	0	0.13±0.01
**17**	301.0387	Quercetin glycoside	0.45±0.07	0.22±0.03
**18**	349.0618	Ethyl gallate derivative	0.12±0.01	0
**19**	285.043	Quercetin	0.26±0.01	0.48±0.07
**20**	271.0627	Naringenin	0.11±0.01	0
**21**	287.2249	Dihydroxyhexadecanoic acid*	0.05±0.01	0.11±0.01
**22**	443.1753	Trihydroxyoctadecenoic acid derivative	0.12±0.01	0.07±0.01
**23**	329.2358	Trihydroxyoctadecenoic acid*	0	0.15±0.01
**24**	663.2948	Dihydroxyhexadecanoic acid derivative*	0.69±0.05	0
**25**	547.2805	Dihydroxyhexadecanoic acid derivative	0.29±0.02	0.11±0.01
**26**	269.0472	Apigenin	0.41±0.04	0.88±0.05
**27**	299.0597	Diosmetin*	0.08±0.01	0
**28**	283.0643	Acacetin*	0.12±0.01	0
**29**	277.1822	Panaxytriol	0	0.49±0.07
**30**	483.3161	Palmitic acid derivative	0.05±0.01	0
**31**	239.0701	Hydroxyflavanone*	0.05±0.01	0
**32**	295.2301	Hydroxylinoleic acid	0.15±0.03	0
**33**	243.1984	Hydroxytetradecanoic acid	0.077±0.019	0
**34**	271.2278	Hydroxyhexadecanoic acid	0.14±0.01	0.39±0.06
**35**	471.3509	Hydroxybetulinic acid	0.06±0.01	0

Peak No.	$[M-H]^-$	Name	Abundance % at 0.5 h	Abundance % at 24 h
**36**	253.2196	Palmitoleic acid (Hexadecenoic acid)	0.02±0.01	0
**37**	279.2351	Linoleic acid*	0.25±0.02	0
**38**	255.2355	Palmitic acid (Hexadecanoic acid)	0.09±0.02	0.22±0.04
**39**	281.2521	Oleic acid*	0.34±0.01	0

* Denotes metabolites that showed significant difference when analyzed using paired t test (p value < 0.05)

Table S2. Metabolites identified in *O. ficus* samples; untreated and treated with ex vivo culture of the human gut microbiome isolated from fecal matter along with their relative abundance

Peak No.	$[M-H]^-$	Rt (sec)	Molecular Formula	Error (ppm)	MS/MS	Name	Class	Untreated *O. ficus* sample	*O. ficus* treated with culture of the human gut microbiome
**1**	195.0504	66	$C_6H_{12}O_7$	3.19	177.01, 133.03	Gluconic acid	Organic acids	++	+
**2**	353.0862	83	$C_{16}H_{18}O_9$	4.53	191.01	Caffeoylquinic acid	Phenolic acid	+	-
**3**	73.0311	97	$C_3H_6O_2$	-8.2	-	Propionic acid	SCFA	-	+
**4**	133.0137	98	$C_4H_6O_5$	4.08	115, 71.01	Malic acid	Organic acid	+	-
**5**	205.0356	101	$C_7H_{10}O_7$	-1.09	191.05, 127, 111.01	Homocitric acid	Organic acid	++	+
**6**	191.0193	110	$C_6H_8O_7$	3.26	171.03, 127, 111, 99, 83.01	(iso)citric acid	Organic acid	+	-
**7**	117.0211	126	$C_4H_6O_4$	-14.98	73.03	Succinic acid	Organic acid	-	+
**8**	173.0091	180	$C_6H_6O_6$	-16.89	129.02, 111.01, 85.03	Aconitic acid	Organic acid	++	+
**9**	129.0216	188	$C_5H_6O_4$	2.56	85.05	Mesaconic acid	Organic acid	+	-
**10**	147.0454	190	$C_9H_8O_2$	-1.67	129.01, 103.03, 85.01	Cinnamic acid	Phenolic acid	++	+

Peak No.	[M-H]$^-$	Rt (sec)	Molecular Formula	Error (ppm)	MS/MS	Name	Class	Untreated *O. ficus* sample	*O. ficus* treated with culture of the human gut microbiome
**11**	255.0557	268	$C_{11}H_{12}O_7$	-0.29	165.05, 119.05, 107.05	Piscidic acid	Phenolic acid	+	++
**12**	117.0557	412	$C_5H_{10}O_3$	0.15	99.02	Hydroxypentanoic acid (hydroxyvaleric acid)	SCFA	+	++
**13**	431.1043	508	$C_{21}H_{20}O_{10}$	-13.72	285.11	Kaempferol rhamnoside	Flavonoids	+	-
**14**	331.0681	527	$C_{13}H_{16}O_{10}$	-3.1	169.01	Galloylglucose	Phenolics	+	-
**15**	473.2102	602	$C_{22}H_{34}O_{11}$	-13.21	301.11, 179.07, 151	Quercetin glycoside	Flavonoids	++	+
**16**	285.0376	616	$C_{15}H_{10}O_6$	10	268.03, 243.03, 195.04, 169.06	Kaempferol	Flavonoids	-	+
**17**	165.0592	643	$C_9H_{10}O_3$	-14.97	147.03, 119.05, 91.01	3-(4-Hydroxyphenyl) propanoic acid	Phenolics	-	+
**18**	423.0918	652	$C_{19}H_{20}O_{11}$	3.5	331.09, 169.01	Galloylarbutin	Phenolics	+	-
**19**	609.1450	673	$C_{27}H_{30}O_{16}$	10.01	447.01, 315.15	Isorhamnetin-O-pentosyl-hexoside	Flavonoids	+	-
**20**	445.0502	674	$C_{20}H_{14}O_{12}$	14.14	301.14, 179.07, 151	Quercetin glycoside	Flavonoids	++	+
**21**	437.1138	695	$C_{20}H_{22}O_{11}$	11.1	331.01, 169.01	Galloylglucose derivative	Phenolics	+	-
**22**	443.1824	709	$C_{17}H_{32}O_{13}$	-12.12	329.23, 133.01, 71.01	Trihydroxyoctadecenoic acid derivative	Fatty acids	++	+

Peak No.	$[M-H]^-$	Rt (sec)	Molecular Formula	Error (ppm)	MS/MS	Name	Class	Untreated *O. ficus* sample	*O. ficus* treated with culture of the human gut microbiome
**23**	541.2636	725	$C_{26}H_{38}O_{12}$	3.39	315.11	Isorhamnetin glycoside	Flavonoids	++	+
**24**	785.2930	746	$C_{29}H_{54}O_{24}$	0.29	315.29	Isorhamnetin glycoside	Flavonoids	+	-
**25**	477.0217	787	$C_{20}H_{14}O_{14}$	12.09	331.01, 169.01	Galloylglucose derivative	Phenolics	+	-
**26**	329.2304	823	$C_{18}H_{34}O_{5}$	8.43	133.01, 71.01	Trihydroxyoctadecenoic acid	Fatty acids	+	++
**27**	533.2029	824	$C_{27}H_{34}O_{11}$	-0.83	329.23	Trihydroxyoctadecenoic acid derivative	Fatty acids	++	+
**28**	301.0327	863	$C_{15}H_{10}O_{7}$	8.86	179.07, 151	Quercetin	Flavonoids	-	+
**29**	235.1736	945	$C_{15}H_{24}O_{2}$	-13.95	217.17, 191.01	Farnesoic acid	Fatty acids	+	-
**30**	271.0641	1015	$C_{15}H_{12}O_{5}$	-10.67	253.15, 209.36, 177.37, 151.01, 119.04	Naringenin	Flavonoids	-	+
**31**	295.2328	1057	$C_{18}H_{32}O_{3}$	-16.65	277.21, 251, 183.13	Hydroxylinoleic acid	Fatty acids	++	+
**32**	281.2513	1156	$C_{18}H_{34}O_{2}$	-9.55	237.03, 171.1	Oleic acid	Fatty acids	+	-
**33**	279.2364	1223	$C_{18}H_{32}O_{2}$	-12.3	237.09, 187.01	Linoleic acid	Fatty acids	++	+
**34**	323.226	1272	$C_{19}H_{32}O_{4}$	-9.83	255.23	Palmitic acid derivative	Fatty acids	+	-

* ++, +, -; reflects the metabolite relative abundance as depicted from the peak abundance data extracted from MS-DIAL, (++) increased abundance, (+) present, (-) absent

Table S3. Metabolites identified in *O. ficus* untreated and treated ex-vivo with actual fecal matter samples along with their relative abundance. Results are expressed as relative percentile (average ± std deviation, n=3) of the total peak areas of identified metabolites.

Peak No.	$[M-H]^-$	Name	Abundance % at untreated sample	Abundance % at treated sample
**1**	195.0504	Gluconic acid*	2.72±0.12	1.26±0.05
**2**	353.0862	Caffeoylquinic acid	0.65±0.01	0.15±0.01
**3**	73.0311	Propionic acid*	0	1.43±0.02
**4**	133.0137	Malic acid*	7.75±0.01	1.81±0.01
**5**	205.0356	Homocitric acid*	2.95±0.51	0.71±0.01
**6**	191.0193	Citric acid*	32.52±0.9	0.56±0.03
**7**	117.0211	Succinic acid*	0.69±0.3	37.96±0.93
**8**	173.0121	Aconitic acid*	19.73±0.08	0.31±0.0
**9**	129.0190	Mesaconic acid*	6.92±0.41	0.11±0.0
**10**	147.0454	Cinnamic acid*	1.37±0.01	0.41±0.0
**11**	255.0511	Piscidic acid*	2.34±0.03	29.76±0.87
**12**	117.0557	Hydroxypentanoic acid*	0.24±0.0	1.99±0.01
**13**	431.1043	Kaempferol rhamnoside*	1.11±0.06	0.03±0.0
**14**	331.0681	Galloylglucose*	0.31±0.01	0
**15**	473.2102	Quercetin glycoside*	1.82±0.03	0.07±0.0
**16**	285.0242	Kaempferol*	0	2.69±0.07
**17**	165.0592	3-(4-Hydroxyphenyl) propanoic acid*	0	2.31±0.02
**18**	423.0918	Galloylarbutin	0.61±0.02	0.03±0.0
**19**	609.1450	Isorhamnetin-O-pentosyl-hexoside	0.21±0.01	0
**21**	437.1138	Galloylglucose derivative*	1.26±0.05	0.15±0.01
**21**	445.0502	Quercetin glycoside*	2.63±0.01	0.18±0.02
**22**	443.1824	Trihydroxyoctadecenoic acid derivative*	1.87±0.05	0
**25**	477.0217	Galloylglucose derivative*	1.05±0.05	0.22±0.01
**26**	329.2304	Trihydroxyoctadecenoic acid*	0.58±0.05	4.22±0.01
**27**	541.2636	Isorhamnetin glycoside*	2.77±0.12	0.26±0.01
**27**	533.2029	Trihydroxyoctadecenoic acid derivative	0.38±0.01	0.15±0.0
**28**	785.2930	Isorhamnetin glycoside*	2.31±0.09	0.45±0.02
**29**	301.0327	Quercetin*	0.13±0.01	3.16±0.02
**29**	235.1736	Farnesoic acid	0.24±0.03	0.03±0.0
**31**	295.2328	Hydroxylinoleic acid*	2.01±0.02	0.26±0.01
**32**	271.0641	Naringenin*	0.11±0.02	3.62±0.02
**32**	281.2513	Oleic acid*	1.11±0.07	0

Peak No.	$[M-H]^-$	Name	Abundance % at untreated sample	Abundance % at treated sample
**33**	279.2364	Linoleic acid	0.83±0.03	0.15±0.0
**34**	323.226	Palmitic acid derivative*	0.92±0.05	0

* Denotes metabolites that showed significant difference when analyzed using paired t test (p value < 0.05)
